# 784. Feet First: A Innovative Curriculum for Podiatry Residents on Infectious Disease using Padlet

**DOI:** 10.1093/ofid/ofad500.845

**Published:** 2023-11-27

**Authors:** Amit M Sharma, Sindhu Srinivas, Jaclyn Denise Wessinger, Elliane Nasser

**Affiliations:** GEISINGER CMC, SCRANTON, Pennsylvania; GEISINGER CMC PODIATRY, SCRANTON, Pennsylvania; GEISINGER CMC PODIATRY, SCRANTON, Pennsylvania; GEISINGER CMC PODIATRY, SCRANTON, Pennsylvania

## Abstract

**Background:**

Infectious Disease Rotation is mandatory for Podiatry trainees. Unfortunately, a standardized Infectious Disease rotation is lacking despite the above requirements. Therefore, we performed a nationwide needs assessment for a dedicated podiatry curriculum for Infectious Disease Elective. We also created a curriculum using Padlet to provide education to our trainees.

Results Table Program Characteristics

**Figure:**
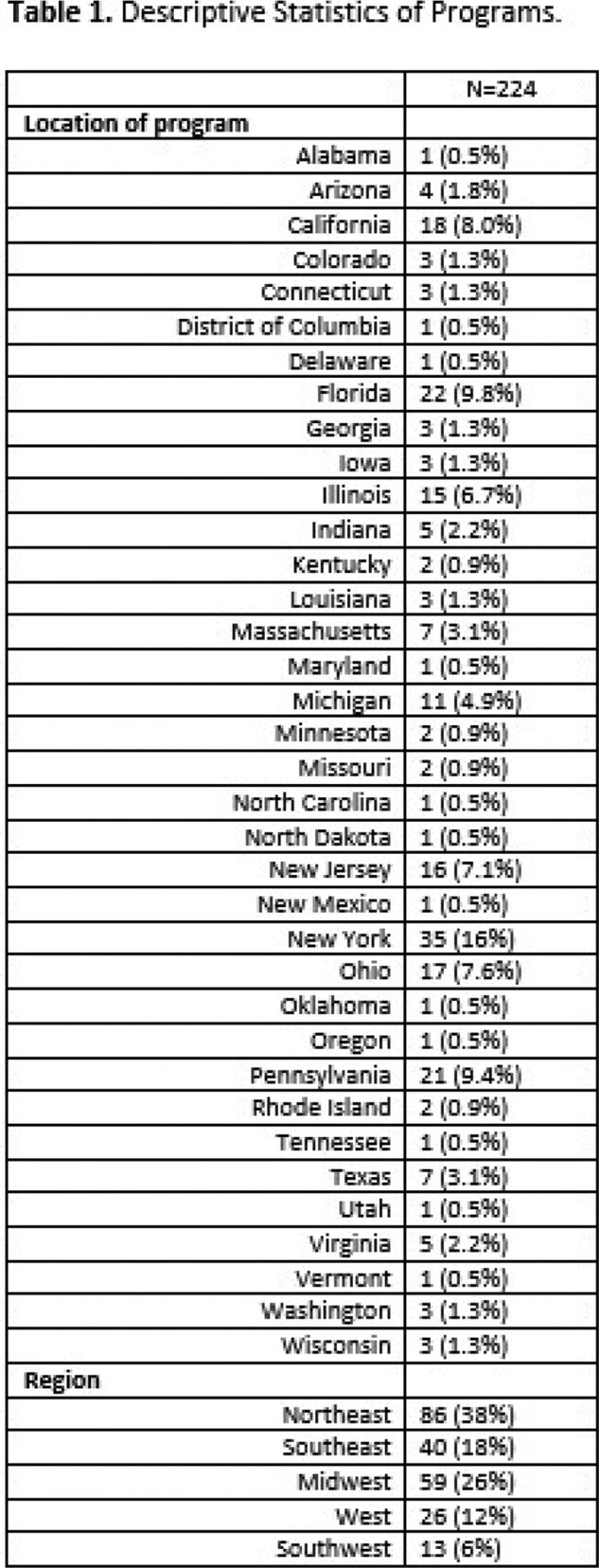

Podiatry Training Program Characteristics across the nation

Table 2 Curriculum characteristics

**Figure:**
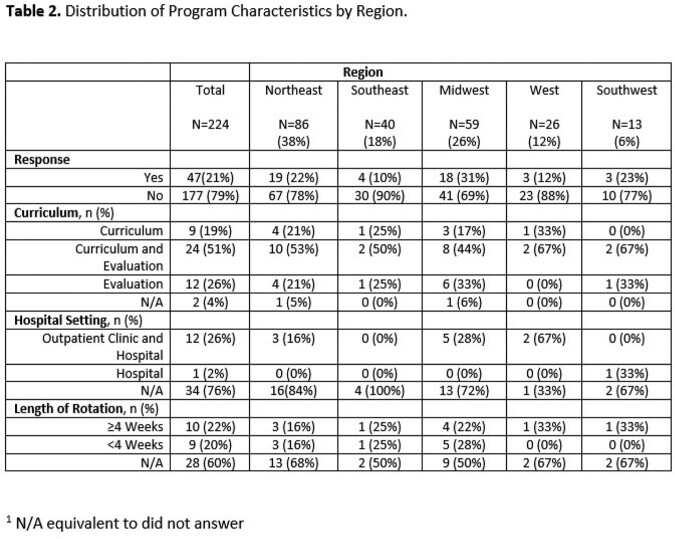

Variation in curriculum

**Methods:**

We performed a nationwide survey to assess the current uniformity between podiatric programs regarding their infectious disease rotation. In addition, each podiatric surgical residency program was surveyed to gather data on its current curriculum and evaluation processes. We surveyed Geisinger North East Podiatry Residents to identify the common topics they wanted to learn from their ID rotation and the learning curriculum best suited to their needs. Finally, we devised a novel approach to medical education that leverages educational technology's power to enhance podiatry residents' learning experience. Using Padlet, a versatile and user-friendly platform, this curriculum offers learners an interactive and engaging learning experience featuring lectures, videos, case studies, quizzes, polls, and assignments.

**Results:**

The Northeast region had 38% of programs, followed by the Midwest (26%), Southeast (18%), West (12%), and Southwest (6%). Common regional themes included identifying nosocomial infections, understanding precautions and quarantine for specific infections, and understanding and treating MRSA, VRE, Clostridium difficile, osteomyelitis, and necrotizing fasciitis among Northeast Programs. In the Western region, programs focused on attitudinal assessments detailing the importance of punctuality, self-study and literature review, accepting criticism, quality improvement, willingness to enhance knowledge and practice, and practicing with professionalism. Based upon the discrepancies in the duration of rotation, breadth of topics covered, and lack of uniformity in teaching techniques, we created a curriculum using the Flipped Classroom method and EdTech tools like Padlet. Through Padlet we hope to combine synchronous and asynchronous educational techniques.

**Figure:**
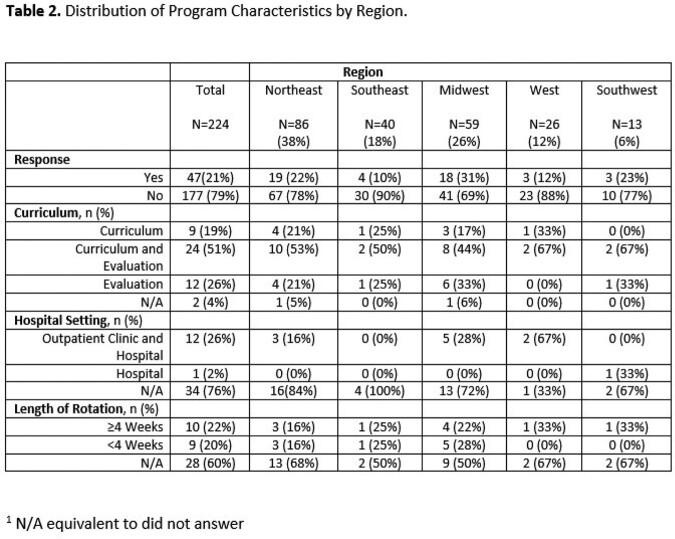

Distribution of podiatry training programs across the United States

**Figure:**
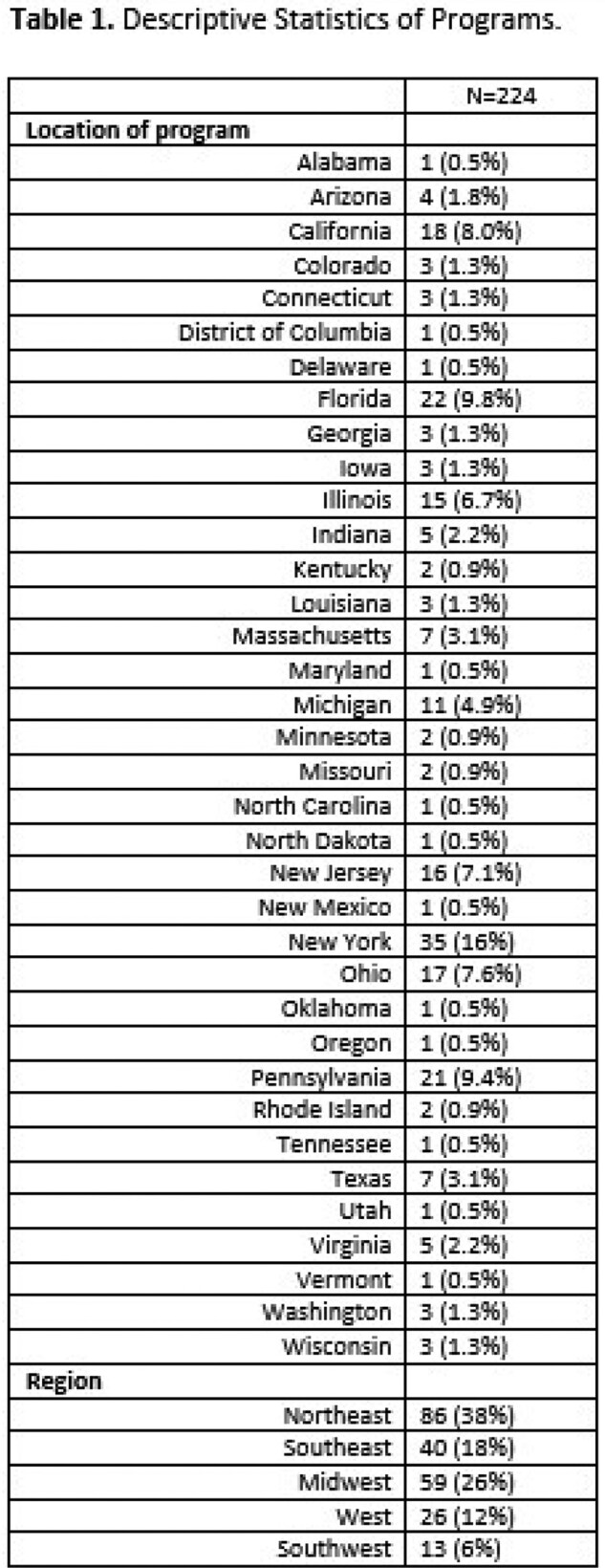

**Conclusion:**

Edtech tools like Padlet can help provide quality education effectively to podiatry trainees.

Padlet Snapshot

**Figure:**
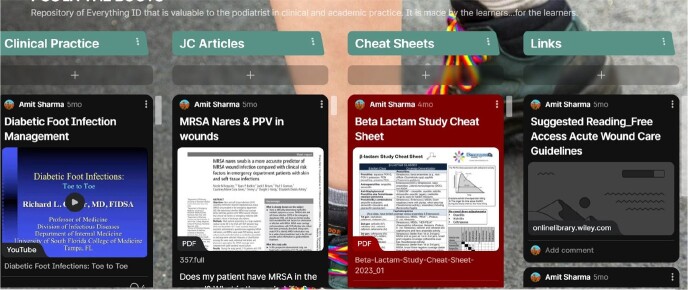

**Disclosures:**

**All Authors**: No reported disclosures

